# Is physical activity a future therapy for patients with Marfan syndrome?

**DOI:** 10.1186/s13023-022-02198-9

**Published:** 2022-02-10

**Authors:** Steeve Jouini, Olivier Milleron, Ludivine Eliahou, Guillaume Jondeau, Damien Vitiello

**Affiliations:** 1 URP 3625-Institut des Sciences du Sport Santé de Paris (I3SP), Université de Paris, 75015 Paris, France; 2 Laboratory for Vascular Translational Science, INSERM U1148, Hôpital Bichat-Claude-Bernard, Université de Paris, Paris, France; 3 Centre National de Référence Pour le Syndrome de Marfan et Pathologies Apparentés, Assistance Publique-Hôpitaux de Paris, Hôpital Bichat, Paris, France; 4 Service de Cardiologie, Centre National de Référence Pour le Syndrome de Marfan et Apparentés, AP-HP, Hôpital Bichat, 75018 Paris, France

## Abstract

**Introduction:**

The international recommendations tend to avoid physical activity (PA) for patients with Marfan syndrome (MFS). However, exceptions have recently been made in the most recent recommendations for these patients, suggesting benefits from doing PA at low intensity only. Furthermore, there is no evidence that moderate aerobic or weight training can worsen the disease symptoms and increase mortality of MFS patients. The present review sums up the work carried out in the field of PA and MFS. The review aims to (1) identify the different types of exercise testing and training protocols and (2) discuss the feasibility and potentially beneficial nature of PA as an innovative way to manage MFS patients.

**Methods:**

The scientific literature was reviewed using the following words: Marfan syndrome, training, physical activity, evaluation, weight training, arterial disease, aneurysms, lung damage, aortic dissection, rupture. A total of 345 studies were prospected and 43 studies were included.

**Conclusions:**

A limited number of studies were done in humans, however one demonstrated the feasibility of the management of MFS patients with PA. There were potential beneficial effects of PA on arterial structures, but this review also showed deleterious effects when PA was conducted at high intensities, corresponding to 75–85% of the maximal oxygen uptake. However, these effects have only been reported in animal studies.

## Introduction

Marfan syndrome (MFS) is a rare autosomal dominant genetic pathology and affects 1:5000 people [1, 2]. About 50% of MFS patients have the presence of a pathogenic mutation in the fibrillin 1 (*FBN1*) gene [3] which represents the causal gene for MFS [4]. The alteration in the expression of *FBN1* causes a significant weakening of the media [5]. More precisely, the disease affects the lamellar units of the medial aortic layer. These medial changes are responsible of the acute aortic dissection which represents the single most important negative event in the life of Marfan patients. The alteration in the expression of *FBN1* also induces damage to the cardiovascular and muscle systems, the eyes, skin, bones and joints [3, 6]. However, arterial disease is the leading cause of death related to MFS if not diagnosed in time, with the consequence of an aneurysm causing dissection and rupture of the aorta.

To date, physical activity (PA) for patients with MFS is still globally not recommended or very limited in intensity (*e.g.* golf, bowling, walking). American recommendations and the authors [7, 8] recommend low-intensity PA because the increased blood pressure during exercise could present a higher risk of aneurysm and dissection of the aorta [9]. Indeed, the outgoing pressures of the left ventricle increase with effort. The ascending aorta (*i.e.* root, sinotubular junction and tubular segment) and the aortic isthmus are much more frequently involved by the disease making this area vulnerable to increased pressures in the vascular system. Finally, Marfan patients with *FBN1* mutations and bicuspid aortic valve exhibit a larger aortic root diameter [10] which is also a vulnerable spot of their vascular system. However, over the past 20 years, doctors have gradually introduced PA in managing patients with MFS [11]. To date, PA that can be offered is still very limited and is, in all cases, low-intensity [12].

The benefits of PA in the general population are widely recognized to date, particularly in reducing cardiovascular risks [13]. For several years, training protocols have been offered to patients with heart failure with impaired or preserved ejection fraction [14–16]. The mortality rate of these patients dropped significantly (*i.e.* the risk ratio for mortality was calculated as 0.65–95% confidence intervals 0.46 to 0.92) after a training program [17, 18]. Patients with coronary artery disease, hypertension, diabetes or obesity have also seen a reduction in mortality following training protocols [19, 20]. In addition, it is recognized that the lack of PA (*i.e.* sedentary lifestyle) is determined by personal, socio-cultural, and demographic barriers [6, 21]. Moreover, in the context of genetic diseases such as MFS, the risk of death from rupture of the aorta is increased from a sedentary lifestyle, especially since the dogma of the rupture of the aneurysm following an increase in PA is already well anchored in the patient’s and clinician’s minds. Thus, the lack of knowledge and data does not allow the healthcare professional to encourage patients with MFS to perform regular PA or to offer specific training programs.

In this context, this literature review aims to identify the different types of exercise testing and training protocols (Table 1) and discuss the feasibility and potentially beneficial nature of PA as an innovative means of management of MFS patients in the future.

**Table 1:** Physical evaluations and training programs in Marfan syndrome

References	Training program	Main results	Conclusion
**Humans**			
Benninghoven et al. [11]	5 times a week for 3 weeks combining ergo cycle training, Nordic walking, and gymnastics 18 MFS patients	(Nordic Walking 666 m P < 0,05% and ergo cycle 0.23 Watt/kg P < 0.001) Improvement of physical capacities	A protocol of physical activity with light intensity seems to have positive effects
Giske et al. [22]	Assessment of lung capacity, the volume of oxygen uptake, and muscle strength	(1) Decrease in strength up to 48% *vs.* control	MFS patients exhibits a decrease in muscle strength. This impairment increase with the speed of exercises
Kolonics-Farkas [23]	Assessment of lung capacity and VO_2_ peak	Decreased lung function The spirometry-specific equation for MFS patients	
Otremski [52]	Correlation between spine and chest wall deformities and lung function	(1) Thoracic kyphosis (mean 19.3°; − 32° to 54°) had a strong positive correlation with FEV1/FVC (r = 0.65; *P* < 0.001) (2) Significant decrease in FEV1/FVC below 80% occurred at kyphosis less than 15° (*P* = 0.004) (3) The chest wall had a strong negative correlation with FEV1/FVC (r = − 0.61; *P* = 0.001)	The deformation of the rib cage and spine impairs lung function
Percheron et al. [25]	Evaluation of the muscle strength of the lower limbs with an isokinetic apparatus	Correlation between muscle strength and muscle mass (r = 0.68; *P* = 0.0048) The strength depended on the movement speed was about 10% lower than the control group with a significant difference of *P* < 0.001	A significant difference in muscle strength was shown for MFS patients
**Animals**			
Gibson et al. [26]	5-month training program with different training intensities from 55 to 85% of VO_2_ max	(1) Number of segments of elastin fibers (2) Length of the elastin fiber (3) Improvement and organization of elastin fibers (4) The compliance of the aorta (5) Reduction of MMP 2–9 at 55% of VO_2_ max (6) Reduction of the aortic diameter (7) Decreased tensions (8) High-intensity exercises (75–85% Vo_2_max) induced aortic aneurysm but this was not observed after moderate intensity exercises (55%-65% Vo_2_max)	Physical activity (forced treadmill or voluntary wheel) dramatically improves the structural architecture of the aorta in a mouse model of MFS (Fbn1^C1039G/+^). The tensions are reduced and allows to stop the dilation as well as to reduce it
Mas-Stachurska et al. [27]	5-month training program on treadmill, 20 cm/s, 12° positive slope for 60 minutes/day, 5 days/week.	(1) aortic root dilation rate blunted by training (2) No improvement of aortic stiffness by training (3) Training did not induce additional structural damage in the tunica media of aorta (4) Regression of left ventricle hypertrophy with training	Training prevented aortic root dilation and partially reversed cardiac hypertrophy in a mouse model of MFS (Fbn1^C1039G/+^)

FEV1, forced expiratory volume during the first one second; FVC, forced vital capacity; VO_2_ max, Maximum oxygen uptake; MMP, matrix metalloproteinase; MFS, Marfan syndrome

## Methods

### Review of the literature

The PubMed, Medline and Google Scholar medical research databases were searched between 1986 and 2021. The terms used were "Marfan syndrome, training, physical activity, evaluation, weight training, arterial disease, aneurysms, lung damage, aortic dissection, rupture." Other studies were consulted, such as recommendations, arterial disease, athlete studies, and the aneurysm relationship, and studies on the effects of cellular proteins (*e.g.* MMP, TIMP, TGF) and the muscle-building type physical activity.

### Selection of articles

**Fig. 1:**
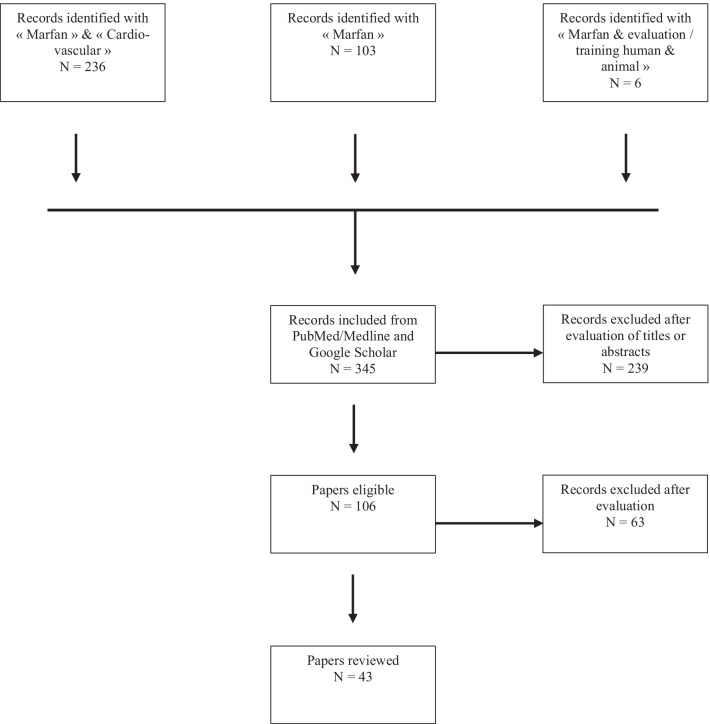
Chart flow of the selection process

The selected articles were all published in scientific literature reviews. Two researchers in exercise physiology and cardiovascular pathology reviewed the different articles. Following this search, duplicate references were removed. We then selected articles through their titles and abstracts. Studies were selected if they were conducted on MFS patients or animal models of MFS and if they performed training or physical activity and physiological evaluations (Fig. 1).

## Results

### Number of selected studies

We identified 345 studies and after reviewing the titles and abstracts, we excluded 236 and included 106. All articles’ books were in the English language. Potentially eligible articles were reviewed in full text. We then grouped the articles according to the subjects or patients studied: “Marfan, arteriopathy, and athletes.” A total of 43 articles were finally included in this review.

### Assessment of pulmonary function and oxygen consumption

To our knowledge, limited studies have evaluated aerobic functional capacity in MFS patients. One study investigated the peak of oxygen uptake (VO_2_ max) and lung function by spirometry [22]. Another study assessed lung function with spirometry only [23].

Giske et al. [22] carried out a spirometry test to measure the forced vital capacity (FVC), the total vital capacity (TVC), the forced expiratory volume during the first second (FEV1), the maximum voluntary ventilation (MVV), the residual volume (RV) and the pulmonary diffusion capacity (DLCO). The authors compared these measurements obtained in male and female patients with predictive values. An increase in TVC of 30% above the mean values was observed. The RV was 2 times higher than normal values. The TVC/RV ratio increased by 40%. The FEV1/FVC ratio had mean values of 75%, which were close to normal values (*i.e.* 80%). However, some subjects exhibited an FEV1/FVC of 68% indicating a slight obstruction of the tracks. The DLCO was within normal limits.

Kolonics-Farkas et al. [24] used the arm span to correct for height as proposed by previous authors, to overcome the rib cage deformities of MFS patients when measuring their height. They then recalculated the FVC and FEV1 based on the arm span using the following equations of the European Coal and Steel Community:

FVC men: 5.76H − 0.026A − 4.34; FVC women: 4.43H − 0.026A − 2.89.

FEV1 men: 4.30H − 0.029A − 2.49; FEV1 women: 3.95H − 0.025A − 2.69.

(H = height replaced here by the arm span, A = age).

The study showed significant differences in FEV1 and FVC, particularly in men, for patients having a surgery involving the ribcage (*i.e. *any cardiovascular intervention or scoliosis) after using the corrected equations. These equations appeared to more accurately represent the patients’ clinical symptoms. In addition, a negative correlation between FVC and the percentage of scoliosis, and between FEV1 and the percentage of scoliosis was demonstrated.

Moreover, Giske et al. [22] also conducted an evaluation of the pulmonary capacities and VO_2_ peak during effort with an evaluation protocol on ergocycle (*i.e.* increase in loads of 25 or 50 W, every 3 min with a pedaling frequency of 60 rpm) in their studied population. The results showed an average VO_2_ peak of 25.3 ml/min/kg for women and 24.4 ml/min/kg for men, and a maximum ventilation/MVV ratio of 60% for women and 48% for men.

Overall, the results obtained in these studies showed a decrease in pulmonary and functional capacity of MFS patients. In addition, patients undergoing surgery showed more significant impairment of their lung function.

### Assessment of muscle function

We identified 2 articles published between 2003 and 2007 which assessed the muscle strength of MFS patients [22, 25]. The number of patients included was 17 (*i.e.* 13 women and 4 men) for the study by Gikes et al. [22], and 21 patients (women only) for the study by Percheron et al. [25].

Giske et al. [22], divided the cohort into 2 groups, 1 control group of 18 healthy sedentary subjects (*i.e. *13 women and 5 men) and 1 experimental group of 17 Marfan patients (*i.e.* 13 women and 4 men). The authors assessed the participants' muscle strength using an isokinetic device that measured the degree-speed torque per second at two different speeds (60°/s and 240°/s) for the extensor and flexor muscles.

At low speeds (60°/s), the strength of flexor muscles of the right leg was slightly decreased by 20% in MFS patients compared to the control group, but without reaching significant difference (*P* = 0.11). Moreover, once the speed was increased, the difference between the two groups increased and became significant (*P* = 0.03).

Percheron et al. [25], divided their cohort into 2 groups. A control group of healthy sedentary subjects and an experimental group of MFS patients. The authors performed various assessments of muscle strength with an isokinetic measurement device (*i.e.* peak force, muscle fatigue, isometric strength, force-speed relationship). The authors also assessed the lean mass and the fat mass of the lower limbs and body.

Regarding the results on muscles of the lower limbs; the authors demonstrated that the muscle mass of the lower limbs (6.4 ± 0.9 kg *vs.* 7.2 ± 0.8 kg, *P* < 0.05) were significantly reduced in MFS patients compared to healthy subjects. They also showed significant correlations between the decrease in muscle mass and the power developed by the quadriceps at 0°/s (r = 0.62, *P* = 0.01), 60°/s (r = 0.62, *P* < 0.01) and 120°/s (r = 0.68, *P* < 0.01) in MFS patients compared to healthy subjects.

### Physical evaluations and training programs in Marfan syndrome

- Physical evaluations in Marfan syndrome
  Several authors have proposed different types of protocols for evaluating the physical components of endurance and strength [22, 25] in MFS patients. However, very few studies have subsequently proposed training protocols or physiological exercise evaluations. To our knowledge, 3 studies [26, 27], including only one performed in humans [11], proposed endurance training protocols after initial physical assessments. A single study on animals proposed different training modes with moderate or high intensities [26].
  These 3 studies were innovative in the management of MFS patients with PA and aimed to observe the influence of training intensity on MFS.
  The training protocols proposed by the authors were mainly carried out to improve aerobic capacity or aerobic power [11, 26, 27].
- Training programs in animal models of Marfan syndrome
  Mas-Stachurska et al. [27] worked on Marfan FBN-1 transgenic mouse model (Fbn1^C1039G/+^). These authors carried out a moderate-intensity training protocol on a healthy sedentary control group (n = 11), a Marfan control group (n = 9), a healthy training group (n = 10) and a training Marfan group (n = 10). Each group was then divided into two sub-groups: physical activity group and sedentary group. The duration of the training protocol was 5 months and the duration of the treadmill training sessions was 60 min at a speed of 25 cm/s.
  In their study, Gibson et al. [26] proposed different training modalities using the same animal model (n = 12). These animals were compared to a control group (n = 16). The training duration was 5 months with 5 sessions per week of 30 min. Running intensities were 65%, 75%, and 85% of VO_2_ max.
- Training program in patients with Marfan syndrome
  In their study, Benninghoven et al. [11] worked with 18 patients diagnosed with FBN-1, including one diagnosed with Loeys-Dietz. The patients were recruited 4 months after their surgery and were classified at NYHA III. Maximal blood pressure and target training heart rate was measured during exercise. The criterion for stopping the effort was set at a systolic blood pressure of 160 mmHg. The authors proposed the following training protocol: (1) a 30-min ergometer training everyday; (2) gymnastics for 60 min four times per week; (3) physical training (weight training) during 60 min three times per week, and (4) Nordic walking 30 min, three times per week. The total duration of the training was 3 weeks or 5 training sessions. In addition, these authors proposed integrating an individual psychotherapeutic dimension into their training protocol, in the form of therapeutic education on "life at work" and "diet."

### Beneficial impacts of training in Marfan syndrome

- Improvement of cardiorespiratory and muscular functions
  In the animal model of MFS (Fbn1^C1039G/+^), the authors notably observed a global improvement in post-training VO_2_ max [26, 27] using different intensities of aerobic training.
  In one study, the authors also assessed cardiorespiratory physical capacity pre-workout without reassessing them at the end of the study [26]. However, throughout the protocol, the authors showed differences in the distances covered by the two groups of animals (*i.e.* control and Fbn1^C1039G/+^). The Fbn1^C1039G/+^ training group had reduced physical capacity and traveled fewer kilometers per day at the start of the protocol compared to the control training group. These differences were gradually reduced throughout the protocol to arrive at a value equal to the one of the control group at the end of training.
  In the other study, the muscle capacity also changed for the trained group [26]. The oxidative state of the muscle fibers had improved without observing an improvement in their density.
  In the sole human study, the authors showed an improvement in the physical capacities of MFS patients following the training program [11]. Significant effects on the parameters of the maximum power developed on an ergometer (+ 0.77 W/kg ± 0.23 W/kg or *P* < 0.001) have been reported, showing an increase of 42% according to their evaluation criteria as well as an increase in walking distance 39% (2356 m ± 666 m or *P* < 0.05).
- Improvement of the elastic fiber structure within the aortic wall
  To analyze elastic fiber structure, the Van Gieson and picrosirius red stains were performed on sections of the aortic wall to observe the structural state of the elastic fibers in the different groups of control and trained animals.
  Gibson et al. [26], showed significant differences in the architecture of the elastic fibers of the ascending aortic wall in the group of sedentary MFS mice compared to the group of sedentary control mice. Regarding the forces and stresses on the arterial walls, the trained mice showed better resistance (≈ 1500 mN * mm^−2^) compared to the control MFS group (≈ 950 mN * mm^−2^) (*P* = 0.009). No difference was observed between the sedentary and trained control groups regarding the length of the elastic fibers. It was shown that the sedentary control group of mice had a longer elastic fiber length than the sedentary MFS group (+ 50%, *P* < 0.001). In contrast, training increased significantly the length of elastic fibers in the ascending aorta of MFS mice (+ 50%, *P* < 0.001) matching the control animals values (sedentary and trained).
  In addition, regarding the number of segments of elastin fibers, no significant difference was demonstrated between the sedentary and trained control groups. The sedentary control group of mice had a significantly smaller number of segments than the sedentary MFS group (− 8, *P* < 0.0001). In contrast, training significantly reduced the number of elastin fiber segments in MFS mice (− 6, *P* < 0.001) matching the control animals values (sedentary and trained).
  Moreover, no significant difference was demonstrated between the sedentary and trained control groups for the percentage of elastin on the total aortic section surface. The sedentary control group of mice had a higher percentage of elastin compared to the sedentary MFS group (≈ + 15%, *P* < 0.0001). In contrast, training significantly increased the percentage of elastin in MFS mice (≈ + 20%, *P* < 0.0001) matching the control animals values (sedentary and trained).
  Finally, the authors evaluated the effect of training intensity on these parameters. Intensities between 55 and 65% of VO_2_ max induced a significant increase of the elastic fiber length (≈ + 80 µm, *P* ≤ 0.0001) and a reduction of the number of segments of elastin fibers (≈ − 10, *P* < 0.01). Higher intensities (*i.e. *75–85%) did not produce better results on these two parameters. Thus, the authors showed significant improvements after a training protocol on the architecture of the elastic fibers of the aorta.
  In their study, Mas-Stachurska et al. [27] showed that regardless of the experimental condition (*i.e.* sedentary or trained), MFS mice, regardless of exercise condition, exhibited a higher number of elastic fiber breaks (≈ 200% in *MFS vs.* wild-type mice, *P* < 0.0001) and percent fibrosis of the aortic vascular wall (≈ 50% more collagen staining in MFS *vs.* wild type mice, *P* < 0.01) compared to wild-type mice.
- Improvement of the aortic structure and vasomotricity
  The two animal studies showed the beneficial effects of training on aortic diameter and rate of aortic dilation. Indeed, it has been shown that training generated a significant decrease in the aortic diameter of MFS mice (≈ − 0.20 mm, *P* < 0.0001) [26] and a decrease in the rate of aortic dilation (≈ − 50%, *P* < 0.01) [27] compared to sedentary MFS mice. The aortic diameter was significantly increased in the sedentary and trained MFS groups compared to the sedentary and trained control groups in one study (+ 0.5 mm, *P* < 0.001) [27] while the other showed only a larger aortic diameter in sedentary MFS mice compared to their control peers (≈ + 0.2 mm, *P* < 0.0001) [26].
  In addition, the authors observed significant differences in the contractility response of aortic vascular smooth muscles between the two groups [26]. They demonstrated a reduction in the maximal contractile force in sedentary MFS mice compared to sedentary control mice. However, after an injection of potassium chloride (KCL), no difference was observed between the two groups. This could signify that there is no alteration in calcium-induced membrane depolarization in aortic smooth muscle cells in MFS mice. Nevertheless, the authors observed an alteration of the contraction and relaxation mechanisms after injecting phenylephrine and L-NG-Nitro arginine methyl ester (L-NAME). Indeed, a decrease in sensitivity to vasoconstriction and vasodilation responses has been reported after injection of phenylephrine and L-NAME.
- Improvement of aortic wall protein level
  The levels of matrix metalloproteinases 2 and 9 (MMP-2 and MMP-9) in the aortic vascular wall were higher in MFS mice compared to control mice [26]. During the training protocol, the authors showed that only the intensity of 55% of the VO_2_ max could significantly reduce the expression of the MMP-2 and MMP-9 proteins (≈ − 75 signal of intensity *P* < 0.035) and ≈ − 40 signal of intensity *P* < 0.014) compared to sedentary MFS groups. The disruption of the aorta’s proteases is one of the leading causes of damage to its wall. Thus, the decrease of MMP-2 and MMP-9 could help regulate the balance of tissue inhibitors of metalloproteinases (TIMPs) and avoid the alteration of the extracellular matrix and architecture of the fibers of the aortic wall.

## Discussion

To our knowledge, this is the first review of the literature describing the effect of light, moderate, and high-intensity PA in the MFS context. In this review, various training protocols performed both in animals and humans and their effects were assessed.

To date, few studies have proposed training protocols with moderate to high intensities as part of MFS care. Two cross-sectional animal studies [26, 27] and one animal model study [28] (which evaluated the structural or cellular aspects following training), and three human studies have been identified. Two studies performed physiological evaluations and have shown decreased strength and impaired aerobic capacity in MFS patients [22, 25]. Only one training protocol has been performed in humans [11], but the proposed exercise intensity was low, and there was little data regarding the assessments of physical exercise capacities. Nevertheless, this latest study has demonstrated the feasibility and safety of training in the context of MFS in humans.

This lack of relevant results on physical capacities was undoubtedly due to the low intensities, proposed during this training protocol, and recommended by international scientific societies. Indeed, these recommendations tend to limit the intensity of exercise sessions regardless of the severity of the pathology (*i.e.* diameter of the aorta).

### Protocols assessing physical capacities in MFS patients

#### Assessment of respiratory capacity (spirometry)

The majority of studies have demonstrated a disruption of the respiratory mechanics of MFS patients. This overall result is partly explained by the deformities of the rib cage commonly found in MFS patients [29, 30]. These results corroborate the study of Streeten et al. [30] showing an impairment of respiratory function in these patients. The respiratory capacities are influenced by the thoracic structures such as the airways, the pleura, the pulmonary parenchyma, and the aorta's surgery [30].

More specifically, concerning respiratory function, it has been shown that MFS patients presented a FVC and an FEV1/FVC ratio greater than 75%, indicating a slight respiratory obstructive component at rest [26].The other parameters measured remained within the normal limits of respiratory function. These results were obtained using the conventional European standards "European Respiratory Society, 1993". This precision is essential because by measuring these same functional parameters with a measurement normalized for the span of the arm (and not for the height of the body), Kolonics-Farkas et al. [23] demonstrated a further increase in pulmonary functional impairment in their cohort of 55 MFS patients compared to the results of Giske et al. [22]. In addition, Kolonics-Farkas et al. [23] observed more severe airway obstructions in post-operative MFS patients compared to non-operated patients (FEV1/FVC = 0.74 ± 0.08 *vs.* 0.80 ± 0.11; *P* = 0.03) and in particular those who had deformities of the rib cage or with scoliosis.

Therefore, it would appear that indexation of respiratory parameters based on corrected body height, obtained from arm span, may be a more realistic method to assess the respiratory capacity of MFS patients, which would allow the training sessions of these patients to be more precisely tailored.

#### Assessment of maximum oxygen uptake during exercise (VO_2_max)

Overall, the various studies have shown a decrease in VO_2_ max in MFS patients from 20 to 30% compared to healthy subjects [22, 31]. These reductions could go up to 50% for MFS women during efforts of intensity close to their theoretical maximum value (*i.e.* 90% of the theoretical maximum heart rate and score > 17 on the Borg scales 6–20).

Furthermore, the ratio of maximal ventilation (VEmax) to MVV, conventionally measured during a stress test, showed similar results between the MFS patients and healthy subjects [22]. These data do not seem consistent with the results obtained by measuring VO_2_ max. This could be explained by the fact that the VEmax is more dependent on the proper functioning of the respiratory mechanics than the VO_2_ max. The latter has not been adjusted for the arm's span as previously suggested. It is therefore difficult to conclude that the stress test for MFS patients matches that of a healthy population.

Thus, it would be important to have objective data obtained by direct measurements of VO_2_ max from MFS patients to precisely assess their oxygenation capacity and thus design the best training sessions for these patients.

#### Assessment of muscle strength

Overall, MFS patients were shown to have significantly decreased muscle strength (*i.e.* quadriceps) compared to healthy subjects. The muscle strength decreased by 48% at a speed of 240°/s in MFS patients compared to the control group (*P* = 0.003) but only in women [22, 25]. It is known that muscle strength is correlated with muscle mass [32, 33]. The latter can be altered by loss of muscle tissue (*i.e.* sarcopenia) or by neuromuscular damage [25].

Giske et al. [22], demonstrated a decrease in force (20% at 60°/s and 48% at 240°/s) in MFS patients compared to healthy subjects. These authors have shown a correlation between isokinetic and isometric strength. Thus, the neuromuscular system also appears to be affected. In addition, Percheron et al. [25], showed a correlation between hamstring muscle mass and force production (r = 0.68; *P* = 0.004) during isokinetic exercises for both groups. However, in isometric exercises, the correlation was only for the MFS group (r = 0.61; *P* = 0.01). Thus, in MFS patients, the loss of strength was greater than in healthy subjects with equivalent lean mass. This last result could be explained by abnormalities of the connective tissue within the skeletal muscles, which would play a deleterious role in the muscle fibers [34]. In fact, in the context of MFS, the insufficient production of FBN-1 by the FBN-1 gene [35, 36] alters the functioning of the endomysium and the perimysium (consisting mainly of FBN-1) and induces a reduction in elastic fibers in muscle tissue and joint tissue. This could contribute to the tendon and joint hyperlaxity [37] responsible for reduced force production by the poorer transmission of forces from the muscle to the joint in MFS patients.

Finally, it has been reported that the assessment of muscle strength in MFS patients is further impaired when speed is increased [22, 25]. Thus, the decrease in muscle strength appears to be dependent on the "muscle-speed" component. This data shows that the force-speed relationship seems to be as much affected as the force itself in MFS patients. In addition, the authors also showed that muscles most affected during isokinetic muscular work were the flexor muscles of the hamstrings compared to the extensor muscles of the quadriceps.

Therefore, it would be important to assess the muscle power of MFS patients to have a more precise idea of the extent to which their muscle capacity has been reached to better adapt to the muscle-strengthening sessions.

### Training protocols for MFS patients

Some authors have performed exercise assessments and proposed training protocols. These studies made it possible to measure functional parameters (*i.e. *lung function, VO_2_, dynamic and static muscle strength) in MFS patients at rest but also under exertion [22, 23, 25]. These 3 studies demonstrated similar impairment of the lung function and decreased muscle strength. However, in the stress test, authors did not investigate prognostic factors such as the oxygen uptake efficiency slope (OUES), the slope of VE/VCO2, or the first ventilatory threshold (VT1). Despite the authors demonstrating a decrease in functional exercise capacity based on an assessment of the VO_2_ peak; there was no specific assessment of the functional capacities of the patients.

There is to date, only one clinical study that has proposed a rehabilitation program in MFS patients [11] and only two studies have been performed in animals [26, 27]. For the first study in MFS patients, the authors observed an improvement in physical capacity, but most importantly; an improvement in quality of life, particularly in the component of physical function of the SF-36 questionnaire following a protocol of low-intensity training. They also showed an improvement in workloads performed on the ergocycle and an increase in the perimeter of Nordic walking, which was achieved during the training sessions. However, the authors did not assess VO_2_ max or the muscle capacity of the patients.

The two studies conducted on animals showed beneficial effects of training on the cellular structures of the aorta. The exercises were only aerobic, in continuous form at 4 different intensities (*i.e.* 55%, 65%, 75%, and 85%) or five times a week [26]. These authors showed that intensities of exertion between 55 and 75% were the most beneficial and that the highest intensity was less interesting on the benefit provided.

### Training and endothelial function in MFS patients

To our knowledge, cellular studies in the MFS patient are extremely rare. However, MFS patients have been shown to have a deregulation of the expression of the gene encoding fibrillin 1. These patients also exhibit structural alteration of the aorta, predisposing it to microdissection, degeneration, and fibrosis of the media [38–40]. The consequences include aneurysmal and ruptured aorta; the leading cause of death in MFS patients [40]. In addition, the increase in pulse wave speed and arterial stiffness index shows an alteration of the aorta [41–43]. Deregulation of the *FBN-1* gene compromises the integrity of the elastic fibers of the endothelium layers [44]. Thus, the mechanisms of the endothelium allowed relaxation and contraction of vascular smooth muscle by calcium-dependent endothelial nitric oxide synthase (eNOS), may be altered in MFS patients [45].

In this context, PA would reduce the alteration of this mechanism by increasing shear forces. It may protect endothelial function by mechanically activating eNOS and thus increasing the bioavailability of nitric oxide (NO) [46, 47]. It is noted that an alteration of the endothelium would inhibit the production of this gas, and conversely, the integrity of the endothelium would be a guarantee of good health. It was shown that the optimal stimulation to release NO would likely be mechanical (*e.g.* increased endothelial wall shear forces). This improvement is only achieved if the training is done at moderate intensities, as has been demonstrated in healthy or diabetic subjects [48, 49].

In summary, animal studies which assessed the impact of training demonstrated an alteration of the endothelium-dependent hyperpolarizing factor (EDHF) [50]. They also demonstrated that this alteration was attenuated following a training protocol of moderate intensity [51]. However, to date, there are no clinical studies showing this improvement in MFS patients.

## Conclusion

Very few studies have been performed on training in the context of Marfan syndrome in humans. The current recommendations tend to restrict the practice of PA to low intensities in MFS patients. To date, two studies conducted in animal models of MFS and one study in human MFS patients have proposed a training protocol. In the clinical study, a very light intensity of exercise was proposed and few physiological variables were investigated. The beneficial effects of PA on arterial structures have only been reported in animal studies. Other studies on MFS patients are warranted to allow the suggestion of exercise recommendation to these patients and improve their QoL in the future.

In addition, evaluations of physiological and physical parameters during exercise are more numerous but remain very heterogeneous and not statistically unreliable. Finally, there is no data on the role of muscle strengthening in the context of MFS.

Currently, there is some evidence that suggests that PA up to a moderate specific intensity, may besafe in MFS patients. There is no strong evidence against the indication of PA at moderate or high intensity in these patients. Considering the present review, we suggest that adapted and personalized PA would be beneficial for MFS patients and could be proposed as a feasible and safe care plan in the future.

## Data Availability

Not applicable.
